# A Review on Equine Piroplasmosis: Epidemiology, Vector Ecology, Risk Factors, Host Immunity, Diagnosis and Control

**DOI:** 10.3390/ijerph16101736

**Published:** 2019-05-16

**Authors:** ThankGod E. Onyiche, Keisuke Suganuma, Ikuo Igarashi, Naoaki Yokoyama, Xuenan Xuan, Oriel Thekisoe

**Affiliations:** 1 Unit for Environmental Sciences and Management, North West University, Potchefstroom Campus, Private Bag X6001, Potchefstroom 2520, South Africa; oriel.thekisoe@nwu.ac.za; 2 Department of Veterinary Parasitology and Entomology, University of Maiduguri, P. M. B. 1069, Maiduguri 600230, Nigeria; 3 National Research Center for Protozoan Diseases, Obihiro University of Agriculture and Veterinary Medicine, Obihiro, Hokkaido 080-8555, Japan; k.suganuma@obihiro.ac.jp (K.S.); igarcpmi@obihiro.ac.jp (I.I.); yokoyama@obihiro.ac.jp (N.Y.); gen@obihiro.ac.jp (X.X.)

**Keywords:** Equine Piroplasmosis, tick-borne disease, epidemiology, ticks, equines, *Babesia caballi*, *Theileria equi*

## Abstract

Equine Piroplasmosis (EP) is a tick-borne disease caused by apicomplexan protozoan parasites, *Babesia caballi* and *Theileria equi*. The disease is responsible for serious economic losses to the equine industry. It principally affects donkeys, horses, mules, and zebra but DNA of the parasites has also been detected in dogs and camels raising doubt about their host specificity. The disease is endemic in tropical and temperate regions of the world where the competent tick vectors are prevalent. Infected equids remain carrier for life with *T. equi* infection, whilst, infection with *B. caballi* is cleared within a few years. This review focuses on all aspects of the disease from the historical overview, biology of the parasite, epidemiology of the disease (specifically highlighting other non-equine hosts, such as dogs and camels), vector, clinical manifestations, risk factors, immunology, genetic diversity, diagnosis, treatment, and prevention.

## 1. Introduction

Piroplasmosis of equids commonly referred to as equine piroplasmosis (EP) is a tick-borne disease of equids (horse, donkey, mule, and zebra) caused by protozoan parasites belonging to the genera *Babesia* and *Theileria* [1]. The two species responsible for causing EP are *Babesia caballi* and *Theileria equi* (Formerly *Babesia equi*). They are both transmitted by tick species belonging to several genera such as *Hyalomma, Rhipicephalus* and *Dermacentor* [2]. Infected animals are carriers of these pathogens for a long period and serve as source of infection to the vectors (ticks), which in turn transmit the parasites to equine hosts [3].

The disease occurs in three forms which could either be acute, subacute, or chronic. Although, equine babesiosis and theileriosis vary, their general clinical signs similarly include fever, anemia, inappetence, oedema, icterus, hepatomegaly, splenomegaly, and death in some cases [4]. EP causes significant economic losses in the equine industry. Economic losses include treatment cost, abortions, loss of activity, and death [5]. EP has several synonyms, it has been referred to as equine malaria [6]; horse tick fever [6]; anthrax fever [7]; equine biliary fever [8]; equine babesiosis; and equine theileriosis [9].

## 2. Historical Perspective

In 1883, Wiltshire first described a fever of the horse which he termed “anthrax fever” caused by *Babesia equi* [10]. Thereafter, it was in 1901, that Arnold Theiler was able to differentiate between horse sickness and equine piroplasmosis [11]. In 1901, Laveran named it *Piroplasma equi* following the description of the intraerythrocytic parasite in the blood of horses [12]. It was also speculated that the disease was seen in a flock of mules in French Indochina and in Sardinia, Italy in a group of horses in the early 19th century [13]. By 1913 and 1917, the western hemisphere reported the first case in a horse in the Panama Canal and in Barbados, West Indies respectively [14]. It was named *Piroplasma equi* based upon morphology and its close resemblance with malaria infection in humans [3,15]. A few years later, it became clearer that two separate pathogens could be responsible for the disease [16].

## 3. Host Range

The host range for EP so far includes horses, donkeys, mules, and zebras [5]. Numerous epidemiological studies have investigated the prevalence of EP among the equids, with more research focus on horses than on donkeys and mules. This may be due to their accessibility and closeness to man. It is likely that there is lack of adequate clinical reports as well as epidemiological research from the zebra as wildlife as compared to other domestic animals which are of greater agricultural and recreational importance. Furthermore, it is much easier to obtain samples from domestic animals than wildlife due to complications of obtaining permits and handling of wild animals as they have to be darted which is also an expensive exercise. 

## 4. Geographical Distribution

**Figure 1:**
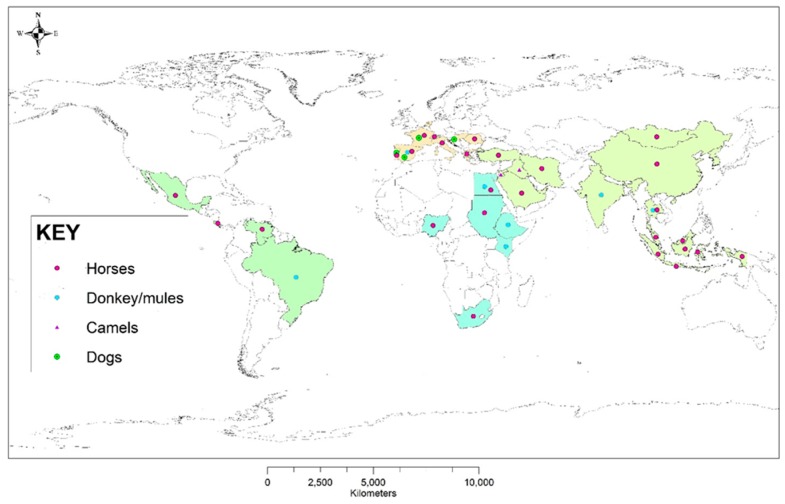
Worldwide distribution of regions where equine piroplasms have been detected or reported across different hosts in the last ten years (2008–2018).

Equine Piroplasmosis is endemic in the tropical and subtropical regions where the tick vectors are known to exist. It has been reported in Asia, South and Central America, Africa, Southern Europe and some parts of the southern USA [17]. The worldwide distribution of equine piroplasms across different regions/countries of the world and notable animal species in which DNA of the parasite has been detected in the last ten years (2008–2018) are presented in Figure 1. Some countries are at risk due to their current non-endemic status of becoming exposed to the pathogen due to frequent movement of equids most especially horses. These countries include Canada, New Zealand, Australia, USA, and Singapore [5,18].

## 5. Basic Biology

### 5.1. Morphology

**Figure 2:**
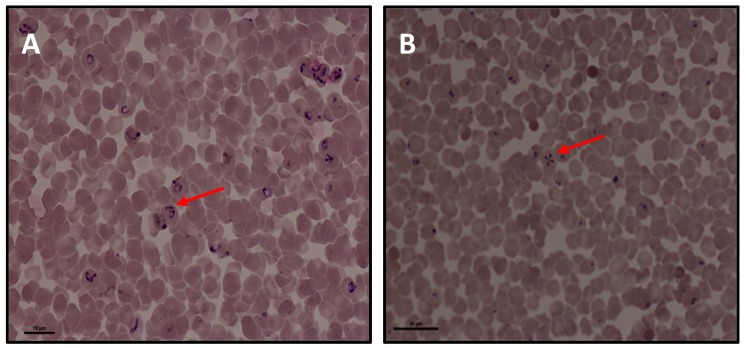
Photomicrograph of Giemsa stained blood smear in Equine Piroplasmosis (EP) parasites prepared from in vitro cultures. (**A**) *Babesia caballi* and (**B**) *Theileria equi*. The arrow on (**A**)indicates the two pear-shaped bodies of *B. caballi* joined at the posterior end; on (**B**) indicates the four shaped merozoites of *T. equi* referred to as the “maltese cross”.

*Babesia caballi* merozoites appear inside the red blood cells as basophilic pear-shaped bodies. The size is approximately 2–5 μm long and 1.3–3.0 μm in diameter [19]. These large two pear-shaped bodies join at their posterior ends (Figure 2A). This is of diagnostic importance peculiar to *Babesia* sp, and by extension *B. caballi* [20]. In *Theileria equi*, the intra-erythrocytic merozoites are smaller, about 2–3 μm long and pyriform, round or ovoid in shape [3]. These four pear-shaped merozoites of *T. equi* form a tetrad called the “maltese cross” in the erythrocytes (Figure 2B). In other words, *Theileria equi* is a small piroplasm whereas *B. caballi* is a larger form [21]. Finally, these two piroplasms are distinguishable by the absence of transovarial transmission of *Theileria* in ticks as well as the lack of pre-erythrycytic cycle in *Babesia* [22].

### 5.2. Life Cycle of Equine Piroplasms

**Figure 3:**
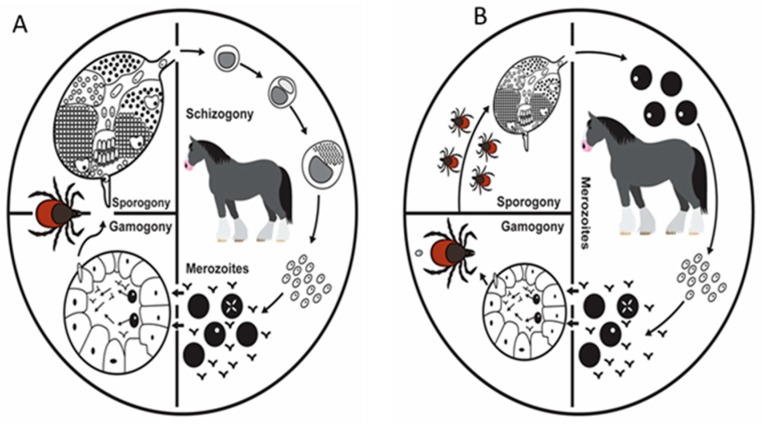
Schematic illustration of the life cycle of *Theileria equi* (**A**) and *Babesia caballi* (**B**) in the vertebrate host and invertebrate vector. Adopted from [23] with few modifications.

The life cycle of a typical apicomplexan has three distinct stages as shown in (Figure 3A,B). These stages occur in the host and ticks and they include; asexual reproduction stage in the salivary glands (sporogony), asexual reproduction stage in the vertebrate host (merogony), and sexual reproduction with the formation and fusion of gametes in the tick gut (gemogony) [23]. In addition, *T. equi* has an additional stage bringing it to four in the life cycle by undergoing schizogony in the peripheral blood mononuclear cells (PBMS).

#### 5.2.1. Theileria equi

Considerable level of variation exists in the life cycle of *T. equi* depending on the species of ticksinvolved [5]. The sporozoites of *T. equi* are injected into an uninfected but susceptible equine species by ticks during feeding via the saliva. On entering the vertebrate host, the sporozoites principally invade the peripheral blood mononuclear cells (lymphocytes) undergo schizogony forming large microschizonts and macroschizonts. These parasites later give rise to merozoites [9]. The released merozoites from schizonts invade the erythrocytes and undergoes merogony forming two pear shaped bodies and in some cases four pear-shaped pyriform bodies which may appear as “Maltese cross” forms [5]. Hemolysis of infected erythrocytes leads to release of merozoites which enter new red blood cells and continue the cycle of replication [9]. Some merozoites transform into rings believed to be gamonts. Following ingestion of the gametocytes in the erythrocytes by a competent tick vector, transformation begins in the midguts of the ticks. It starts with nuclear division followed by formation of protrusions and finally ray bodies (Figure 3A) [23]. Approximately 4–6 days later, ray bodies divide and form microgamates and macrogamates. Fusion of these gametes leads to the formation of zygotes (sexual replication). Kinetes are formed inside the zygote which invades the epithelial cells of the midguts into the hemolymph and infect tick salivary glands type III cells [5,24]. In the cells of the salivary glands, sporonts, sporoblast, and sporozoites are formed. 

#### 5.2.2. Babesia caballi

The life cycle of *B. caballi* is initiated following feeding of infected ticks on an uninfected but susceptible equine host. The tick vectors inoculate sporozoites which are mature via saliva into the vertebrate host. The sporozoites of *B. caballi* invade the red blood cells (erythrocytes) where they are converted into trophozoites which undergo growth and cleave into two pear-shaped merozoites [5]. Merozoites multiply and infect new red blood cells and the cycle is repeated [25]. 

When a competent but naïve tick feeds on an infected blood ingesting parasitized red blood cells, some of the parasites (merozoite) are destroyed in the midgut of the ticks. Nevertheless, some merozoites survive forming a small round of bodies that float within the tick gut. The parasite then undergoes gametogenesis. This begins with nuclear division and subsequently leads to the formation of ray bodies without protrusions (Figure 3B) [23]. The ray bodies are in principle considered to be microgametes and macrogametes. Fertilization results in the fusion of the microgametes and macrogametes to form some diploid zygotes which invade the epithelial cells of the tick midgut and subsequently develop into ookinetes [23]. The ookinetes of *B. caballi* undergo two asexual cycles of multiplication [9]. The first is the invasion of the tick tissues like muscle fibers, hemocytes, the Malpighian tubules, and ovaries of the ticks [26] (Figure 3B). The eggs are infected and subsequently, the larvae. In the invaded tissues, the ookinetes undergo the second asexual multiplication, leading to the production of secondary ookinetes which invade the salivary glands. Sporogony takes place in the salivary glands and the sporozoites mature there. The small pyriform bodies (sporozoites) that are produced in the salivary glands of any of the life stages of the ticks (larvae, nymphs and adults) are passed to the next generation of ticks and are infective to naive animals if any of the ticks forms are feeding [27]. 

## 6. Transmission of Equine Piroplasms

**Figure 4:**
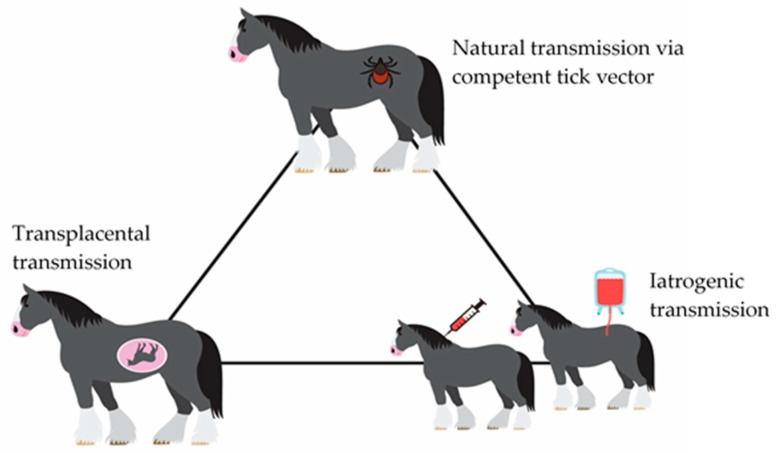
Possible transmission routes of equine piroplasmosis.

The confirmed vectors for EP are competent ticks belonging to three genera, *Dermacentor, Rhipicephalus,* and *Hyalomma* [2]. Nevertheless, recent evidence shows that other genera such as *Ixodes, Haemaphysalis,* and *Ambloyomma* are capable of biologically transmitting the EP parasites under both natural and experimental conditions [20]. Trans-placental transmission from pregnant mares to fetus has been reported [28,29,30], in most cases leading to abortion [30]. Recently, evidence of trans-placental transmission was reported in mules and was particularly associated more with *T. equi* raising concern about the importance of this pathogen in breeding of mules [31]. Transmission by this means is of little epidemiological significance. Furthermore, mechanical or iatrogenic transmission by contaminated needles and syringes, blood transfusion, and surgical instruments has also been reported [30]. A schematic presentation of the possible transmission method for EP parasites is presented in Figure 4. Experimental infection of the parasites to mammalian hosts can be carried out through intravenous or subcutaneous routes in addition to tick transmission [24]. 

## 7. Immunity in Equine Proplasmosis

The equine host immune response to infection due to equine piroplasms infections is complex and multifaceted. Once an animal is infected with either *T. equi* or *B. caballi*, protective immunity is developed against the disease because the animal becomes a carrier of the pathogen [5,32]. Cross-immunity between both parasites does not exist. Therefore, an equid can be infected with either or both pathogens simultaneously [33]. The host innate immunity plays a vital role during babesiosis, [34] but the precise role of the innate immune system like the macrophages, neutrophils, and natural killer (NK) cells in the control of the parasite is not known [32]. Nevertheless, nitric oxide produced by macrophages could be the mechanism which the immune system utilizes during experimental infection with *B. caballi* [35]. Organs of the body most notably the spleen play a vital role in the elimination of most hemoprotozoan parasites including piroplasms. An intact equid with a spleen can overcome and survive an infection with *T. equi* whereas splenectomized horses easily succumb to the disease developing parasitemia as high as 80% [36].

Adaptive immunity could be vital for the successful protection against EP mostly by *T. equi* despite the important role of the spleen and innate immunity. These could be due to the valuable role of cell-mediated immunity to other hemoprotozoan pathogens like *Theileria parva* and *B. bovis* but the precise function of cell-mediated immunity to equine Piroplasmosis is yet to be fully understood [37,38]. Experimental infection of foal with *T. equi* in a spleen-intact foal with severe combined immunodeficiency (SCID) was unable to control parasitaemaia due to *T. equi* [37]. This was attributed to the absence of matured T and B lymphocytes in SCID foals thereby making it difficult to mount antigen specific immune responses [37]. It was therefore concluded that the spleen alone in the absence of specific immune responses (Adaptive immunity) is unable to control infection with *T. equi*. Furthermore, equine peripheral blood lymphocytes have the capacity to proliferate in the presence of *T. equi* lymphoblastoid transformed stimulator cells [39]. Findings from their study demonstrated that peripheral blood lymphocytes from immune animals inhibited the growth of lymphoblasts containing schizonts *in vitro*. In addition, a delayed cutaneous hypersensitivity and inhibition of leukocytic migration was observed in donkeys infected by *T. equi* [40]. Observations from the above-mentioned experiments give further credence to the fact that a correlation exists between cell-mediated and protective immunity to *T. equi* infection in equines. Furthermore, a direct relationship seems to exist between the antibody titers and parasitemia in horses [41]. Horses infected with *T. equi* produces antibodies against equine merozoite antigens (EMAs) which are proteins highly expressed on merozoites [37]. 

Seven unique equine immunoglobulin G have been discovered in the equine genome. Their roles in antibody response during infection and persistence is yet to be elucidated [32]. Immunoglobulin IgGa (now IgG1) and IgGb (now IgG4 and 7) antibody levels increase during acute stage of infection with *T. equi* whereas IgG(T), majorly IgG5 and to a lesser extent IgG3 levels increase during chronic infection when the level of parasitemia is low [41]. These antibodies are detected in the first instance at about 7–11 days following experimental infection and reach a climax at 30–45 days after infection [42]. These antibody subclasses are detectable at the chronic stage of the disease.

Information on protective immunity against *B. caballi* is lacking as compared to *T. equi* infected horses. In endemic areas, maternal antibodies in colostrum protect the young foals from infection in their first 1–5 months. In most cases, they can be protected until 9 months of age [2]. When the antibody titer declines, the foal becomes susceptible to infection and most horses in nations with endemic stability of the infection are infected by two years of age [32]. Finally, it appears that cell-mediated immunity and cytokines play a crucial role in the immune response to *B. caballi* infection. In experimental *B. caballi* infection in horses, it was observed that nitric oxide (NO), tumor necrosis factor alpha (TNF–α) and other cytokines enhanced protective response when produced in optimal amount that neutralized the parasites [35].

## 8. Clinical Manifestations

### 8.1. Clinical Signs

Following tick transmission, the incubation period is approximately 15 and 20 days for *T. equi* and *B. caballi* respectively [4]. In endemic areas, there are no pathognomonic clinical signs of infection [43]. Manifestation of clinical disease can take different forms which could either be peracute, acute, subacute or chronic [5]. Sudden onset of clinical signs which could lead to death occurs in the peracute form while as in the acute form, signs includes fever, inappetence, malaise, and peripheral oedema. Gastrointestinal involvement such as colic followed by diarrhea has also been observed [3]. Clinical signs in foals are nonspecific such as weakness and decreased suckling [3]. Donkeys usually manifest the chronic form of EP rather than horses. Signs of chronic infection are usually nonspecific and manifested as weight loss, as well as poor performance and condition [3,44]. In addition, abortion and neonatal death have been reported following intrauterine infections [43]. 

### 8.2. Haematological Changes during Equine Piroplasmosis

Most equids regardless of the clinical form of infection exhibit some degree of anemia which is the result of hemolysis of infected erythrocytes. At the initial stage, it is normocytic followed by macrocytic as the number of reticulocytes increases in circulation [45]. The erythrocytic indices (Packed Cell Volume (PCV), Haemoglobin (Hb). and Red Blood Cells (RBC) counts) in most of the studies following natural infection were found to decrease in infected animals compared with uninfected animals in both horses and donkeys [44,46]. Erythrocytic parameters such as Mean Capsular Volume (MCV), Mean Capsular Haeemoglobin (MCH). and Mean Capsular Haemoglobin Concentration (MCHC) were variable with notable increases in the concentration of MCH and MCHC in most of the studies [44,46]. Thrombocytopenia has been observed in several studies of clinical EP [47,48]. 

### 8.3. Serum Biochemical Changes

Biochemical changes associated with infection with EP have been investigated in several studies involving equids. Fluctuations in these parameters are affected by a whole range of factors such as exercise, nutrition, weather, hydration status, concurrent presence of other infectious or non-infectious agents, and the general state of health of the study subjects most especially under natural conditions. This could be responsible for the various variations reported by different researchers. A significant decrease in the total protein and an increase in bilirubin, aspartate aminotransaminase (AST), gamma-glutamyltransferase (GGT), creatinine kinase and alkaline phosphatase (ALP) was observed in racing horses clinically affected by babesiosis [49]. In another study, Kumar et al. [38] reported changes in AST, alanine aminotransferase (ALT), ALP and albumin/globulin ratio during drug efficacy studies in splenectomised donkeys experimentally infected with *T. equi* infection.

Furthermore, Camacho et al. [50] reported that infected horses with EP (*B. caballi* and *T. equi*) in Spain had higher serum bilirubin, Urea, AST, GGT, and Lactate Dehydrogenase (LDH) concentrations. They attributed the higher increase in the bilirubin concentrations to be an indication of hemolytic anemia which is a characteristic of EP. In another study, increased bilirubin, alteration of serum phosphorus and hypoalbuminemia was observed in equine piroplasmosis [47]. Conclusively, elevations in the serum levels of liver enzymes are attributed to reduced flow of blood to the liver leading to centrilobular necrosis and changes in the concentration of phosphates and iron could be attributed to erythrocytic metabolism [47]. 

### 8.4. Gross Pathological Findings

Death usually occurs in the peracute form due to multi organ dysfunction which may be unconnected with systemic intravascular coagulation [3]. Other findings include subcutaneous edema most especially of the eyelids and other subserosal tissues [23]. Splenomegaly, hepatomegaly, hemorrhages of the epicardial and endocardial tissues, discolored and large kidneys and lymph nodes, and congestions of the lungs have been reported.

### 8.5. Histopathological Findings

Lesions observed following histopathology includes centrilobular necrosis of the liver and microthrombi within the liver and lungs [30]. In addition, renal tubular necrosis with hemoglobin casts. Finally, proliferation of reticuloendothelial cells in the lymph nodes, lungs, kidney, and liver have been observed [45].

### 8.6. Pathophysiology of Anemia in Equine Piroplasmosis

**Figure 5:**
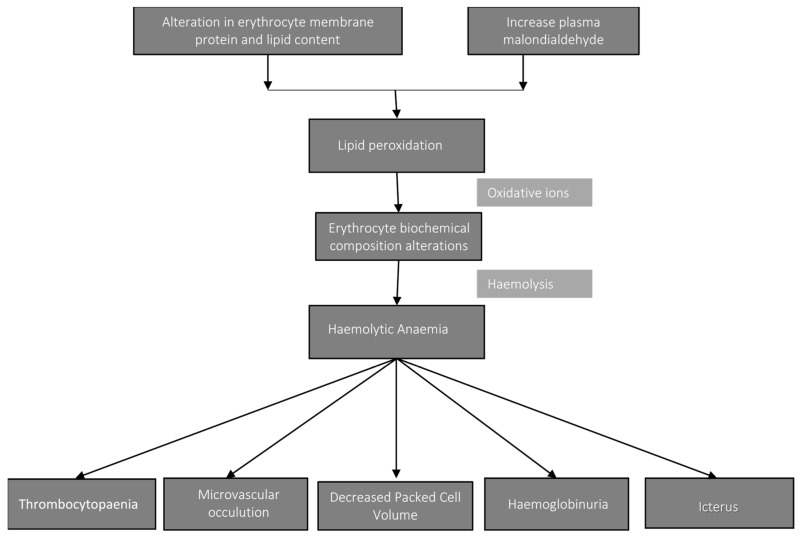
Schematic presentation of pathophysiology of anemia in equine piroplasmosis. The flowchart illustrates the sequence of events that culminates in anemia and other pathological sequellae.

Hemolytic anemia is one of the consequences of the infection of the erythrocytes by the blood parasites *B. caballi* and *T. equi*. Conformation alteration in the erythrocyte membrane protein and lipid content lead to reduced microvascular blood flow [32]. Also, there is increase in the plasma levels of malondialdehyde during parasitemia. This results in the accumulation of oxidative ions, a product of lipid peroxidation which subsequently alters the erythrocyte biochemical composition leading to erythrocyte lysis (hemolysis) [36]. Continuous hemolysis further leads to a cascade of events such as decreases in the packed cell volume (PCV), hemoglobinuria and icterus. These events have been observed in *T. equi* infection. While as in the case of *B. caballi* infection, microvascular occlusion has been observed due to clumping of parasitized erythrocytes [5]. Thrombocytopenia of varying degrees and longer clothing times have been associated with *T. equi* and *B. caballi* infection [51]. The pathogenesis of thrombocytopenia remains uncertain but it is postulated to be either through immune-mediated destruction or splenic sequestration [32]. A schematic presentation of the pathophysiology is presented in (Figure 5).

## 9. Ticks as Vector of Equine Piroplasmosis

Ticks play a vital role in the stable maintenance and natural transmission of the piroplasms involved in equine piroplasmosis. The global distribution and spread of EP (*B. caballi* and *T. equi*) is dependent on the availability of competent tick vectors that are capable of transmitting these pathogens to equines (horse, donkey, mule, and zebra). Various attempts have been made in recent times to list tick species that have been implicated as competent vectors responsible for transmission of EP parasites in the equidae [5,20,52]. Most recently, Scoles and Ueti [20] listed 33 species of ixodid ticks belonging to six genera as competent vectors responsible for EP. The list is likely not exhaustive as most studies have only focused on a small portion of ticks with strong association to equines. Ticks belonging to the genera *Hyalomma, Dermacentor, Rhipicephalus* are the three well known vectors that have been incriminated as the biological vector for *T. equi* and *B. caballi* [2,53]. Other genera of ticks including *Amblyomma, Haemaphysalis* and *Ixodes* are suspected but not confirmed [20]. 

*Babesia caballi* is transmitted trans-ovarially while *T. equi* is transmitted either trans-stadially or intra-stadially [23]. In trans-stadial transmission, the nymphal stages of the competent ticks acquire *T. equi* infections through either an acutely or chronically infected equid during a blood meal and after molting and transfer to a new host, infect a naïve host successfully [24]. While as in intra-stadial transmission of *T. equi*, adult ticks pick up the infection during a blood meal and after moving to a new host, transmit the parasite successfully [24]. When a female tick acquires *B. caballi* parasite during a blood meal and vertically transmits the pathogen to the next generation it is called trans-ovarial transmission. Naïve horses acquire the infection during a blood meal from an infected tick. These infected ticks can transmit the parasites to naïve animals over several generations without re-infection from an infected host [24]. *Hyalomma excavatum*, *H. anatolicum*, *Rhipicephalus bursa*, *Boophilus microplus,* and *Amblyomma cajennense* are known vectors for *T. equi* and have been reported from different parts of the world [54,55,56,57] although *Amblyomma cajennense* has been proven to be an intrastradial transmitter of *T. equi* [58]. Vectors known to transmit *B. caballi* include *Dermacentor nitens*, *Dermacentor albipictus, Hyalomma truncatum* and *Rhipicephalus evertsi evertsi* [11,59,60,61]. 

## 10. Risk Factors Associated with Equine Piroplasmosis

The positivity of equids to piroplasmosis has been assessed in numerous studies to be connected with several risk factors. Risk factors such as age, sex, breed, animal species, castration status, presence or absence of ticks, location, origin, and activity have been investigated in several studies [1,43,62]. Better understanding of the risk factors for EP is crucial for the identification of the population at risk which will aid in the formulation of better control measures when equids are moved from diseased areas to a disease free zone [63]. Risk factors could either be intrinsic host related or extrinsic/environmental related. 

### 10.1. Intrinsic Risk Factors

Various intrinsic risk factors have been attributed to be responsible for higher or lower prevalence of piroplasmosis in the equid. These risk factors include the following:

#### 10.1.1. Equine Species

Horses, donkeys, mules, and zebra are known equine species susceptible to EP. Susceptibility to infection with EP is more prevalent in horses than donkeys with regards to *T. equi* [1,62]. Other researchers reported that mules were more susceptible to EP than horses in Brazil [64], Spain [65] and Greece [43]. Kouam et al. [43] attributed the higher prevalence in mules to the frequent outdoor activities of the mules where they are involved in daily transportation of wood from the forest. This was further linked to their extended exposure to pasture which increases their likelihood to bites by ticks.

#### 10.1.2. Age, Gender, and Breeds

Generally, the prevalence of EP has been found to increase with increasing age. Infection with *B. caballi* decreases with age while higher prevalence of *T. equi* has been reported compared with *B. caballi* with increasing age. The increase may be unconnected with increasing immunity with rising age with resultant elimination of the parasite as in the case of *B. caballi* [18]. This has been confirmed in several experimental and epidemiological studies were *T. equi* persist and *B. caballi* are eliminated with increasing age [66,67]. A positive correlation was reported between host age and *T. equi* infection only [43,66]. Finally, some studies did not find age to have influence on the prevalence of EP [67].

Furthermore, male and female have shown different susceptibilities to protozoan infection. These variations have been attributed to levels of sex hormones [68]. Epidemiological data from different studies around the world are contradictory on the effect of sex on the infection rate. Some studies show female to be more infected than males to *T. equi* [66,67]. Some other researchers found no correlation between seropositivity and sex to both *B. caballi* and *T. equi* [69,70]. Finally, others found males to be more infected than females with regards to *B. caballi* infection [1]. In some studies, breeds were found to have influence on the rate of seropositivity to EP [67]. It was observed that the Italian rapid heavy draft farm horse (TPR) breed of horse in Italy was more exposed to both *B. caballi* and *T. equi* individually than the other breeds while the cross-breds were more exposed to *B. caballi* as well as double infection [67].

#### 10.1.3. Host Activity and Castration Status

Host activity has been shown in some studies to influence the positivity of horses to EP [43]. Horses used for sports where found to have lower prevalence due to reduced exposure to tick infestation due to better parasite control measures [67]. Equids kept in farms are more likely to be exposed to competent tick vectors which may increase the risk of infection to EP [71]. 

Castrated male equids had lower tick burden as compared to non-castrated ones and were three times more likely to be infected with *T. equi* as compared with non-castrated animals [66]. Although, experimental findings in mice proved otherwise, where elevated levels of testosterone increased the chances of infection with piroplasms [72] and increased their susceptibility to tick infestations [73], although, sex-specific husbandry techniques may play a role in the above observation. Therefore, this underlines the importance of verifying laboratory findings with epidemiological studies [74]. 

### 10.2. Environmental or Extrinsic Risk Factors

The environmental or extrinsic risk factors associated with the prevalence of EP are as follows: 

#### Altitude, Grazing and Climatic Factors

High altitude of over 1500 to 3200 m has an influence on the species diversity of collected ticks. This is evident by the collection of over 90% of *D. nutalli* in Gobi steppe in Mongolia [75]. In another study, the results were contradictory where the prevalence of EP due to *T. equi* reduces as the altitude decreases. In the latter, the findings were attributed to climatic interference in the tick cycle as the altitude increases [64]. The chances of direct contact with ticks are increased when animals are used for farm work and graze with other domestic or companion animals. This has been reported as a risk factor associated with equine piroplasmosis by several authors [65,67,76]. This risk has been estimated in equines to be two and eleven times likely for *T. equi* and *B. caballi* respectively and only nine times for both [67]. Variation in climatic condition among different areas has an influence on seropositivity to EP [67]. This is because climatic condition influences the distribution of tick vectors and their periods of activity. It was therefore inferred that orographical and geographical factors could play a role in influencing the distribution of vectors and species responsible for EP. Furthermore, it was observed that horses reared in certain areas had more contact with *B. caballi* and *T. equi* than horses raised in other areas [67]. Finally, management practices as related to animal husbandry have influence on the rate of positivity to EP [77]. 

## 11. Epidemiology of Equine Piroplasmosis

In the last five decades, extensive epidemiological studies have been carried out in different continents of the world on the epidemiology of EP and their associated risk factors. The list of never ending studies continues in the quest to ascertain the spread of these parasites among the equine population. EP is endemic in many parts of Europe [78,79], Africa [48,80], Asia [56,81], and America [82,83] where suitable tick vectors are present.

Due to the variations in the sensitivity of different diagnostic tests used in different epidemiological studies, different prevalence observations have been reported from various areas in the world. Furthermore, the difference in the prevalence from different epidemiological studies could also be due to risk factors that promulgate the infection such as the presence and abundance of competent tick vectors, activity of the host, management practices, and the effectiveness of vector control programs if any. In the majority of the studies in endemic countries, the prevalence of *T. equi* is generally higher than that of *B. caballi* [43,48,65,77,84]. This could be explained by the fact that infected equids which survive the infection completely eliminate *B. caballi* from their blood circulation after about 4 years whereas *T. equi* infested equids remain life-long carriers [18]. Geographical distribution of reported epidemiological studies of EP using different diagnostic techniques in different continents in the last 10 years in horses and donkeys/mules are summarized in Table 1 and Table 2 respectively.

## 12. Genetic Diversity of Equine Piroplasmosis Parasites

The use of PCRs has helped overcome the challenges faced in the detection of low parasitemia due to EP parasite infections. It has also made it possible for genetic characterization to be carried out [108]. For the detection of piroplasms in equines, different gene targets have been employed such as 18S rRNA gene [109], EMA-1 [110], and *β*-tubulin gene [111]. 

Nuclear rRNA genes have been shown to provide appropriate targets to assist in the identification of species [112]. Despite this advantage, several different researchers have reported sequence heterogeneity within *B. caballi* and *T. equi* in Africa [113,114], the Middle east [1], Asia [98], Europe [115,116], North America [117], and South America [101]. Early studies on genetic diversity undertaken in Spain and Greece detected two genotypes named *Babesia*/*Theileria* and “like forms” [116,117].

Only recently, Manna et al., [108] reported the existence of different genotypes for both *T. equi* and *B. caballi*, three different groups namely (A, B, and C) and (A, B1, and B2) respectively. In addition, Qablan et al., [1], identified genotype C of *B. caballi*. Bhoora et al., [113] in South Africa reported two groups (A and B) with 11 samples clustering in group A and only two in group B. Other studies reported four groups [114,116,118] and three (3) *T. equi* group (A, B, D) in Jordan [1] and five *T. equi* groups (A, B, C, D, and E) in Switzerland [119]. One interesting observation in most of the above reviewed studies was the concomitant occurrence of various genotypes within a sampled population of equids [1,113,114]. Furthermore, results obtained in Italy postulated that group A was prevalent in symptomatic animals while B was more prevalent in asymptomatic subjects [106]. These findings agree with other researchers [117,119] on the prevalence of group A and B genotypes in symptomatic and asymptomatic animals respectively. Additionally, Bhoora et al. [113] observed more variation in the genotypes of *T. equi* in zebra than horses in South Africa. 

Conclusively, genetic diversity plays an important role in influencing disease transmission, pathogenicity and the degree of sensitivity of the diagnostic system [108]. 

## 13. Notes on the Detection of Equine Piroplasms in Non-Equine Hosts

According to Criado-Fornelio et al., [120], “the universe of Piroplasms is still expanding” at genetic level and host range. Both *B. caballi* and *T. equi,* which are responsible for EP in equines, were traditionally thought to be highly host-specific [121]. Recent epidemiological studies have revealed the detection of these equine piroplasmid species in different hosts raising concerns about their host specificity [122,123,124]. 

Molecular techniques with sequencing of the PCR products have enabled the detection of these piroplasmids in other animal species outside the traditional equids (horses, mules, donkeys, and zebras) such as in dogs [120,122,125,126] and camels [123,127]. Recently, DNA of *T. equi* was amplified in a South American tapir (*Tapirus terrestris*) [128]. The epidemiological reports are summarized in Table 3. 

## 14. Diagnosis of Equine Piroplasmosis

### 14.1. Microscopy and in Vitro Culture

Microscopy is one of the readily available techniques for the identification of parasites within the erythrocytes if they are present. This can be achieved with the use of a thin blood smear stained with any of the Romanosky’s stains most especially 10% Giemsa solution [5]. It is most useful during the acute phase of infection with EP. Nevertheless, the smear should be thoroughly examined requiring a skilled operator to avoid false-negative results [4]. This is so because the parasitemia level remains low even during the acute phase of infection. *In vitro* cultures of blood from suspected EP parasite infection are also useful if parasitemia is low in the animal [130]. *In vitro* culture has been employed successfully for the identification of *B. caballi* and *T. equi* in blood samples in carrier equids [131]. The only setbacks of this technique are that it is cumbersome, expensive, and inconsistent. 

### 14.2. Serology

Several serological assays have been developed to increase diagnostic sensitivity in equids chronically infected with *B. caballi* and *T. equi* piroplasms. Some of these diagnostic assays include the compliment fixation test (CFT), enzyme-linked immunosorbent assay (ELISA), the immunochromatographic test (ICT), Western blot, and indirect immunofluorescence assay [32,132]. Each of these serological techniques has some advantages or disadvantages. This depends on specificity, sensitivity, simplicity, and cost. Furthermore, it has been suggested that at least two different tests should be used [133]. Serological results that are highly positive may be definitive while negative results may not necessarily preclude infection. Antibody titers have been shown to have no direct relationship with parasitemia [134]. Nevertheless, serological assays are most useful in large scale epidemiological studies.

### 14.3. Complement Fixation Test (CFT)

This technique remains the serologic test of choice for the confirmation of EP. In the recent past, it used to be the officially acceptable test recommended by OIE for the screening of horses before entering EP free nations [5]. This assay can detect antibody titers from day 8 post infection and these titers begin to decline after 2–3 months’ post infection. A negative result can be confirmed within 3–15 months and 24 months respectively as in the case of *B. caballi* and *T. equi* infected horses [18,134]. Some of the setbacks in the use of this technique are that false-negative results are not uncommon and the antigens need to be produced in large amount with cross reactivity between antibodies to the two parasites (*T. equi* and *B. caballi*) [18,135]. CFT should not be considered for the diagnosis of chronic cases due to the presence of immunoglobulin GT (IgGT) antibodies. 

### 14.4. Indirect Immunofluorescent Antibody Test (IFAT)

The IFAT is more sensitive as compared to CFT. It also has high specificity and it is a supplementary test when the results obtained by CFT are inconclusive [4,18]. The principle of this test is based upon the fluorescence of the labeled antibodies of the test sera against the bound antigen on a glass slide. A strong fluorescence under ultraviolet light is considered positive at a dilution equal or greater than 1:80. In experimental infected horses with *B. caballi* or *T. equi*, antibody responses occur at about 3–20 days post infection and can still be detected during the chronic period of infection [5]. Test sera remain positive to IFAT for a longer duration compared with CFT. Test sera must be diluted to increase specificity when using IFAT. However, IFAT is time consuming, it is highly subjective most especially in interpreting the fluorescence, and requires a large quantity of antigen [18].

### 14.5. Enzyme-Linked Immunosorbent Assay (ELISA)

Antibodies to parasites responsible for EP can be detected using ELISA, although, cross-reactivity is not uncommon [18]. The use of cELISA, for the detection of *T. equi* infection was developed by Knowles et al [136] in 1991 using *T. equi* EMA-1 and monoclonal antibodies that are specific [136]. It is the most sensitive test for the detection of chronic infection due to *T. equi*. EMA-1 is a protein found on the surface of erythrocytes infected specifically with *T. equi* and possesses an epitope that is conserved worldwide and is immunodominant [40]. Infected horses with *T. equi* can be detected with cELISA at day 21 after experimental infection and 5 weeks post tick transmission [136]. Production of recombinant protein and associated monoclonal antibodies facilitates the standardization of this test thereby increasing sensitivity compared with other serological assays [41]. In an epidemiological study using this test, it identified 25% more positive samples for *B. caballi* compared to CFT [137]. Finally, in 2004, the OIE approved the cELISA as one of the regulatory tests for the screening of horses for both *B. caballi* and *T. equi* before international transport to non-endemic nations [5].

### 14.6. Immunochromatographic Test (ICT)

The ICT is based on lateral/capillary flow technique whereby antibodies or antigens are mounted on a paper strip or nitrocellulose membrane [138]. The ICT assays for EP have been developed and evaluated on serum samples from horses [132]. However, the use of these EP ICT assays has been restricted to research use and has not been used at reference diagnostic laboratories. 

### 14.7. DNA Based Diagnostic Techniques

Detection of pathogens using the polymerase chain reaction (PCR) is more sensitive than any other methods and is best for diagnosis of animals in chronic infection with EP. It has been extensively used in research settings for the detection of both *B. caballi* and *T. equi* infections in equids and tick vectors. It augments diagnostic results and therefore is regarded as a supplement to microscopy and serology. Variations of PCR including conventional PCR, nested PCR, real-time PCR, and reverse line blot hybridization have been employed in various epidemiological investigation of EP [80,96,113,118]. Generally, PCR technique is straightforward and is becoming more affordable. Another DNA based diagnostic technique is loop-mediated isothermal amplification (LAMP) which uses four or six primers, is rapid, highly sensitive, and cost effective [139]. Alhassan et al. [130] developed LAMP assays targeting EMA1 and Bc48 genes of *T. equi* and *B. caballi* respectively and further reported that they have superior detection efficiency as compared to *in vitro* cultures and conventional PCR [130]. The use of LAMP for large scale epidemiological studies still needs to be explored. However, these DNA-based diagnostic tests are still not used for diagnostic purposes by resource poor countries where EP is endemic.

## 15. Treatment

Treatment of equids with EP is a means of reducing the clinical signs and case fatality rate. Infection with *T. equi* are more difficult to treat than *B. caballi* infections [32]. A few drugs have shown considerable efficacy in eliminating *B. caballi* and *T. equi* infections [135]. However, clearance of these pathogens does not have any considerable impact especially in endemic countries as life-long immunity is conferred with chronic but non clinical infection [32] but in non-endemic countries, treatment with the intention for total clearance of the pathogen is desirable [32]. The use of imidocard dipropionate has shown considerable efficiency in eliminating both *T. equi* and *B. caballi* parasites during the chronic stage of infection [140]. According to Frerichs and Holbrook, [141], the recommended dosage for treatment in the case of *B. caballi* is 2 mg/kg in two doses at 24 h interval and 4 mg/kg in four doses at 72 h interval for *T. equi* [142]. All treatment should be through the intramuscular route. The use of other drugs has been attempted with results that are variable and inconsistent. These drugs include amicarbalide [2], diminazine aceturate [32], and oxytetracycline [47]. Although not in use, the following compounds have been reported to be effective in inhibiting the growth of EP parasites *in vitro*, clotrimazole, ketoconazole, and clodinafop-propargyl [143]; artesunate, pyrimethamine, and pamaquine [144], as well as nitidine chloride and camptothecin [32,145].

## 16. Prevention of Equine Piroplasmosis

Successful control and prevention of EP is dependent on the proper control of the vectors. This remains a big challenge more especially in tropical and subtropical regions. The use of acaricides has been widely adopted for the control of the vector while prophylactic treatment of horses has also been adopted by the locals in some endemic areas. This method has helped to a great extent in reducing the burden of tick infestation on susceptible animals. In the short term, tick control on horses and donkeys has been achieved using classes of acaricides such as organophosphates, pyrethroids, and amidines [23]. 

Isolated cases have been reported mostly linked to blood contaminated equipment or due to blood transfusions [25,45]. Therefore, adequate care must be taken to prevent unnecessary and preventable transmissions. Furthermore, attempts to use vaccination to induce immunity to EP have been attempted in donkeys [38], but no commercial vaccine is available. On a general note, control of vector borne infections can only be successful through routine surveillance and adequate knowledge of vector distribution, vectorial competence and habitat [32].

## 17. Concluding Remarks

It is becoming glaringly obvious that many disease pathogens that affect the health of livestock, human, wildlife and even plants globally are transmitted via specific arthropod vectors. Unarguably, the first arthropods to be recognized as vectors of disease to humans and animals were ticks [146]. Tick-borne pathogens are dangerous to the health of livestock and by extension, public health most especially during the tick season. The pathogens in ticks are maintained in complex cycles and could be transmitted to other domestic and or companion animals including humans as incidental host. Therefore, the spectrum of tick-borne diseases infecting humans and domestic animals is on the rise giving concern to veterinarians and physicians [147].

Recent evidence has proved otherwise from the earlier belief that piroplasms were host specific. *Babesia caballi* and *Theileria equi* which were thought initially to only specifically affect equines but recently DNA of these piroplasms has been detected in dogs and camels [122,127]. The list may continue to increase and these pathogens may even jump to humans due to increasing interactions between equids, humans, and ticks. Therefore, a holistic one health approach is the way to go as far as the control and prevention of increased distribution and host range. Furthermore, the discoveries of causative agents responsible for EP in other host outside the equids were made possible through the use of molecular techniques including sequencing. These underscore the need for a better and robust institutional framework to equip, train, and re-train manpower more especially in the developing resource poor countries. The review on the prevalence of EP in different continents and countries of the world (see Table 1) indicates the contrasting differences in technological application and use in different parts of the world. The developing countries still rely on the use of microscopy which is unreliable especially during chronic infection of equids with EP. This is unacceptable in view of the fact that the world is currently into the 4th industrial revolution which has brought about advanced artificial intelligence, molecular and nanotechnology which is changing all facets of life including diagnostics.

Additionally, the complex interwoven relationship contributing to the increase in arthropod vectors and vector-borne disease patterns is unequivocally linked to climate change. The distribution of ticks is intertwined to climatic factors such as humidity, temperature, vegetation cover, and land use among others. Climate change related to global warming is expected to influence the distribution of vectors and reservoir hosts which will ultimately affect the epidemiology of the vector-borne pathogens [148]. In recent times, mathematical modeling has no doubt gained momentum which can be used as a tool to address biological and ecological details on the transmission of diseases. Modelling studies are required in order to predict possible future outbreaks and spread of the disease. A multidisciplinary initiative involving the veterinary sector, ecologists, climatologists, and the economic sector is needed to ensure proper control of EP as it affects animals which are used on a daily basis as draught animals, for transport, tourism as well as recreational activities (including horse racing) which all contribute to the economies of countries. 

Finally, there is an urgent need to search for alternative biomolecules that will assist to overcome the current problems of acaricide resistance due to inappropriate application and use and treatment failures because of the development of pathogen resistance to the available drugs. This has a resultant perpetuation of infection in the ticks for naïve equids to become infected. Furthermore, veterinarians with expertise in equine medicine are few, more especially in resource poor countries where local quacks without the requisite training are involved in the treatment and management of diseases of equines. Effects of global warming and climate change on the distribution of tick vectors will need to be monitored as this might result in further distribution of EP into new territories. With the 4th Industrial revolution on the horizon, developing countries need to step up on the use of modern technology for control of EP, by this era the majority of EP endemic countries should be using a combination of conventional microscopy, molecular diagnostic techniques, geographical information system (GIS), drones, mathematical modelling to mention but a few techniques for the monitoring of control strategies against EP vectors and the disease itself.

## Figures and Tables

**Table 1:** Prevalence of equine piroplasmosis in horses in endemic continents and countries using different diagnostic methods (2008–2018).

Diagnostic Technique	Year	Continent	Country	Sample Size	Prevalence (%) (*B. caballi* & *T. equi*)	Reference
**Microscopy**	**2008**	Africa	South Africa	129	0 and 3.0	[80]
	**2014**	Africa	Nigeria	240	6.0 and 94.0	[85]
	**2014**	Africa	Nigeria	400	83.3 and 11.1	[86]
	**2016**	Africa	Nigeria	156	2.7 and 10.3	[87]
	**2014**	Asia	Iran	100	0 and 5.0	[56]
	**2016**	Asia	Malaysia	306	22.2 and 16.9	[88]
	**2017**	Asia	Turkey	27	0 and 4.8	[89]
**IFAT**	**2008**	Africa	South Africa	99	51.5 and 97.9	[80]
	**2016**	Africa	Egypt	88	17.0 and 23.9	[48]
	**2010**	Asia	United Arab Emirates	105	10.5 and 33.3	[90]
	**2012**	Asia	Saudi Arabia	241	7.5 and 10.4	[91]
	**2014**	Asia	Iran	100	2.0 and 48.0	[56]
	**2014**	Asia	Thailand	63	11.1 and 3.2	[92]
	**2012**	North America	Mexico	248	27.4 and 45.2	[82]
	**2010**	Europe	Switzerland	689	1.5 and 4.4	[93]
	**2012**	Europe	Netherlands	300	3 and 1	[78]
	**2013**	Europe	Hungry	324	NA and 31.8	[94]
	**2017**	Europe	Spain	3100	21.0 and 44.0	[95]
**PCR**	**2008**	Africa	Sudan	131	0 and 25.2	[77]
	**2008**	Africa	South Africa	99	0 and 12.1	[80]
	**2013**	Africa	Tunisia	104	0.9 and 11.5	[96]
	**2016**	Africa	Egypt	88	19.3 and 36.4	[48]
	**2013**	Asia	Mongolia	250	42.4 and 6.4	[97]
	**2013**	Asia	Jordan	288	7.3 and 18.8	[1]
	**2013**	Asia	Korea	224	0 and 0.9	[98]
	**2014**	Asia	Thailand	63	0 and 0	[92]
	**2014**	Asia	Iran	100	0 and 45.0	[56]
	**2016**	Asia	India	160	0 and 11.9	[81]
	**2017**	Asia	Turkey	125	0 and 8.8	[89]
	**2018**	Asia	Indonesia	235	2.1 and 6.4	[84]
	**2015**	North America	Costa Rica	130	20 and 46.2	[83]
	**2017**	North America	Cuba	100	25 and 73	[99]
	**2013**	South America	Venezuela	136	4.4 and 61.8	[100]
	**2017**	South America	Brazil	36	60 and 38.5	[101]
	**2012**	Europe	Netherlands	300	NA and 1.6	[78]
	**2013**	Europe	Hungry	324	NA and 15.1	[94]
	**2014**	Europe	Romania	178	2.2 and 20.3	[102]
	**2016**	Europe	Italy	263	10.3 and 70.3	[79]
**ELISA**	**2008**	Africa	Sudan	158	4.4 and 63.5	[77]
	**2016**	Africa	Egypt	88	14.8 and 0	[48]
	**2016**	Africa	Nigeria	252	4.4 and 65.6	[87]
	**2010**	Asia	United Arab Emirates	105	15.2 and 32.5	[90]
	**2011**	Asia	Korea	184	0 and 1.1	[103]
	**2012**	Asia	Jordan	253	0 and 14.6	[76]
	**2013**	Asia	Mongolia	250	51.6 and 19.6	[97]
	**2014**	Asia	China	1990	51.2 and 11.5	[104]
	**2014**	Asia	Thailand	63	1.6 and 0	[92]
	**2016**	Asia	Malaysia	306	63.1 and 51.3	[88]
	**2016**	Asia	India	160	0 and 73.1	[81]
	**2018**	Asia	Indonesia	235	0.4 and 1.7	[84]
	**2009**	South America	Brazil	538	69.6 and 26.6	[54]
	**2013**	South America	Venezuela	694	23.2 and 14.0	[96]
	**2017**	South America	Brazil	39	7.7 and 43.5	[101]
	**2015**	North America	Costa Rica	130	69.2 and 88.5	[83]
	**2010**	Europe	Greece	524	1.1 and 9.2	[43]
	**2016**	Europe	Italy	673	8.9 and 39.8	[79]
	**2017**	Europe	Spain	108	6.5 and 53.7	[95]
**CFT**	**2009**	South America	Brazil	582	54.6 and 28.5	[54]
	**2015**	Europe	France	443	12.9 and 58.0	[63]

Abbreviations: NA—not available in the text, CFT—Compliment Fixation Test, IFAT—Indirect Florescent Antibody Test, cELISA—Competitive Enzyme-linked Immunosorbent Assay, ELISA—Enzyme Linked Immunosorbent Assay, PCR—Polymerase Chain Reaction.

**Table 2:** Prevalence of equine piroplasmosis in donkeys/mules in endemic continents and countries using different diagnostic methods (2008–2018).

Diagnostic Technique	Year	Continent	Country	Equine Spec (Donkey/Mules)	Sample Size	Prevalence (%) (*B. caballi* & *T. equi*)	Reference
**Microscopy**	2013	Africa	Ethiopia	Donkeys	393	1.8 and 12.2	[105]
**cELISA**	2015	Africa	Kenya	Donkeys	314	0 and 81.2	[106]
	2016	Africa	Egypt	Donkeys	51	0 and 18.0	[48]
**ELISA**	2014	Asia	Thailand	Mules	177	3.4 and 7.3	[92]
	2016	Asia	India	Donkeys	20	0 and 80.0	[81]
	2013	Europe	Spain	Mules	56	32.1 and 66.1	[65]
	2013	Europe	Spain	Donkeys	53	17.0 and 47.2	[65]
	2012	South America	Brazil	Donkeys	88	NA and 73.9	[107]
**IFAT**	2013	Africa	Ethiopia	Donkeys	395	13.2 and 55.7	[105]
	2016	Africa	Egypt	Donkeys	51	22.3 and 26.6	[48]
	2014	Asia	Thailand	Mules	177	2.8 and 10.7	[92]
	2015	Europe	Italy	Donkey	138	40.6 and 47.8	[44]
	2012	South America	Brazil	Donkeys	88	93.2 and NA	[107]
**PCR**	2016	Africa	Egypt	Donkeys	51	18 and 38.8	[48]
	2014	Asia	Thailand	Mules	177	0 and 1.7	[92]
	2016	Asia	India	Donkeys/mules	20	NA and 35.0	[81]
	2012	South America	Brazil	Donkeys	88	20.5 and 31.8	[107]
	2015	Europe	Italy	Donkeys	134	17.4 and 3.4	[44]

Abbreviations: NA—not available in the text, CFT—Compliment Fixation Test, IFAT—Indirect Florescent Antibody Test, cELISA—Competitive Enzyme Linked Immunosorbent Assay, ELISA—Enzyme Linked Immunosorbent Assay, PCR—Polymerase Chain Reaction.

**Table 3:** Reported prevalence on detection of DNA to *Babesia caballi* and *Theileria equi* in dogs and camels in endemic continents and countries using PCR.

Year	Continent	Country	Species	Diagnostic Method	Sample Size	Prevalence (%) (*B. caballi* & *T. equi*)	Reference
2003	Europe	Spain, Portugal and France	Dogs	PCR	10	NA and 40.0	[120]
2009	Europe	Croatia	Dogs	PCR	81	1.3 and 1.3	[129]
2010	Europe	France	Dogs	PCR	166	0.6 and 19.0	[122]
2019	South America	Paraguay	Dogs	PCR	284	NA and 0.3	[126]
2012	Asia	Jordan	Camels	PCR	100	60.0 and 40.0	[123]
2015	Asia	Iraq	Camels	PCR	38	39.5 and 23.7	[127]

Abbreviations: NA—not available in the text; PCR—Polymerase Chain Reaction.
